# HIV care continuum outcomes among recently diagnosed people with HIV (PWH) in Washington, DC

**DOI:** 10.1017/S0950268823000043

**Published:** 2023-01-30

**Authors:** Maria Jaurretche, Morgan Byrne, Lindsey Powers Happ, Matt Levy, Michael Horberg, Alan Greenberg, Amanda D. Castel, Anne K. Monroe

**Affiliations:** 1 Department of Epidemiology, George Washington University Milken Institute School of Public Health, Washington, DC, USA; 2 Westat, Rockville, MD, USA; 3 Mid-Atlantic Permanente Research Institute, Kaiser Permanente Mid-Atlantic States, Rockville, MD, USA

**Keywords:** AIDS, ART initiation, care continuum, cohort, HIV, late diagnosis, retention in care, test and treat, viral suppression

## Abstract

The *Ending the HIV Epidemic* initiative aims to decrease new HIV infections and promote test-and-treat strategies. Our aims were to establish a baseline of HIV outcomes among newly diagnosed PWH in Washington, DC (DC), a ‘hotspot’ for the HIV epidemic. We also examined sociodemographic and clinical factors associated with retention in care (RIC), antiretroviral therapy (ART) initiation and viral suppression (*VS*) among newly diagnosed PWH in the DC Cohort from 2011–2016. Among 455 newly diagnosed participants, 92% were RIC at 12 months, ART was initiated in 65% at 3 months and 91% at 12 months, VS in at least 17% at 3 months and 82% at 12 months and 55% of those with VS at 12 months had sustained VS for an additional 12 months. AIDS diagnosis was associated with RIC (aOR 2.99; 1.13–2.28), ART initiation by 3 months (aOR 2.58; 1.61–4.12) and VS by 12 months (aOR4.87; 1.69–14.03). This analysis contributes to our understanding of the HIV treatment dynamics of persons with recently diagnosed HIV infection in a city with a severe HIV epidemic.

## Introduction

Washington, DC, has been among some of the urban centres with the highest incidence and prevalence rates of HIV in the United States (US) [1]. In the latest annual surveillance report for HIV, the District of Columbia (DC) Department of Health (DC Health) reported a prevalence rate of 1.8% in 2019 [2], which continues to exceed the World Health Organization (WHO)'s threshold of 1% for a generalised epidemic [3]. In response to high rates of HIV in the nation's capital, DC Health and the National Institutes of Health (NIH) launched a multipronged initiative to improve efforts to curb the local epidemic in 2009 [4]. The DC Cohort study, a longitudinal cohort study enrolling people living with HIV (PWH) receiving care in Washington DC, was conceived as one of the pillars of this partnership. The overall objectives of the cohort are to characterise HIV outcomes in DC and to improve the quality of care for PWH [5, 6].

The National HIV/AIDS Strategy (NHAS) uses the HIV care continuum as a functional tool and strategy to improve HIV treatment outcomes nationwide [7]. The HIV care continuum model describes the sequential and essential steps of HIV clinical care [8], namely that PWH that have been tested, linked to care, prescribed antiretroviral therapy (ART), and are virally suppressed [9]. Viral suppression (*VS*) is the ultimate goal of the care continuum as it improves clinical outcomes for PWH [10, 11] and reduces transmission rates as individuals who are suppressed are unable to transmit HIV [9, 12–14]. While DC Health surveillance data indicates that newly diagnosed HIV cases steadily decreased from 405 in 2015 to 217 in 2020, annual infection rates continue to exceed the national average [2]. Applying the HIV care continuum model to persons with newly diagnosed HIV in Washington DC can help improve our understanding of treatment dynamics in this population, identify gaps in the test-and-treat approach, gauge the impact of current evidence-based interventions and policies, and align with the NHAS's *Ending the HIV Epidemic (EHE): A Plan for America* [15]. The objective of this analysis was to examine sociodemographic and clinical factors associated with retention in care (RIC), ART initiation and *VS* among newly diagnosed PWH in the DC Cohort.

## Methods

### Study population and data sources

The DC Cohort study is a city-wide multisite longitudinal cohort study of PWH seeking care in 15 major community-based, hospital/academic and government clinical sites in Washington, DC. At the time of this analysis, there were 14 participating outpatient sites: 9 hospital-based clinics and 5 community-based clinics [5]. All participants provided written informed consent for inclusion in the cohort. The study is approved by the Institutional Review Board (IRB) at George Washington University and by participating sites with their own IRB. Sociodemographic and clinical data are abstracted from the clinics' electronic medical records. They are biannually linked to the DC Health HIV/AIDS surveillance system, which provides additional HIV laboratory results and supplementary clinical data from hospitals and clinics where participants seek care. HIV and AIDS diagnosis dates are also confirmed or added when missing [16].

The DC Cohort is a prospective cohort. We performed secondary data analysis of existing cohort data. For this analysis, we included participants enrolled in the DC Cohort between 1 January 2011 and 31 March 2016, unrestricted by age, who were diagnosed with HIV within 12 months of the enrolment date and had at least one year of follow-up data (hereafter referred to as people who were recently diagnosed with HIV). We examined an additional 12 months of HIV RNA laboratory results after one year of diagnosis to assess sustained *VS*.

### Care continuum outcomes

We used a diagnosis-based care continuum model [8] as we included only diagnosed individuals enrolled in the DC Cohort. We used the Institute of Medicine's definition for RIC, which was defined as having at least two HIV-related medical encounters at least 90 days apart in the 12 months post-enrolment in the DC Cohort [17]. Since medical encounter data is only available after enrolment in the DC Cohort, as it is not routinely captured in local HIV surveillance data, RIC could only be estimated after consent to study participation. ART initiation was defined as being prescribed any antiretroviral medication during two time points: 3 and 12 months following the HIV diagnosis date. In the DC Cohort, data collection includes ART initiation date, which can be a date prior to DC Cohort enrolment. VS was defined as at least one HIV RNA laboratory result <200 copies/μl [18] within the time frame of interest. This measure was assessed at two time points: 3 and 12 months following HIV diagnosis date. Frequency of HIV RNA assessment is at the discretion of the individual providers. HIV RNA labs were eligible to be assessed at 3 months if they were taken between DC Cohort enrolment and the end of month 3. The window for the 12-month HIV RNA was DC Cohort enrolment until the end of month 12.

The source of data for this information could be either local HIV surveillance data or data sent to the DC Cohort by the clinic. For all participants who achieved VS within one year of follow-up, an additional 12 months of HIV RNA labs were evaluated from the first date of VS to determine if VS was sustained. Again, these values could come from either local HIV surveillance data or clinic data. Participants with all HIV RNA values suppressed during the additional 12 months of follow-up were considered to have achieved sustained VS. Based on data availability, RIC was measured in the 12 months post-enrolment in the DC Cohort. However, ART initiation and VS were measured in the 12 months post-HIV diagnosis.

### Covariates

Additional measures of interest were collected at enrolment. These consisted of sociodemographic factors and HIV-related characteristics, including state of residence, HIV transmission risk, insurance (public *vs.* private), housing and employment status, site of care types (community *vs.* hospital-based clinic), smoking (ever *vs.* never), substance use (ever *vs.* never), alcohol abuse (ever *vs.* never), AIDS diagnosis, nadir CD4 at enrolment, HIV RNA laboratory results, HIV duration and year of diagnosis. AIDS diagnosis was defined as having a diagnosis of an AIDS-defining illness, a low CD4 count result of less than 200 cells/μL, or a record of an AIDS diagnosis date prior to enrolment [19].

### Statistical analysis

The number of participants not retained and retained in care was calculated. The association between RIC and covariates were assessed among categorical variables using proportions (%) and median (IQR) continuous variables, testing for significance using Chi-square (Fisher's exact test for small sample sizes) for frequencies and Wilcoxon rank-sum tests medians, respectively.

Separate multivariate logistic regression (LR) models were used to explore the association between covariates and six care continuum outcomes: (1) RIC (2) ART initiation at 3 months (3) ART initiation at 12 months (4) VS achieved at 3 months (5) VS achieved at 12 months (6) VS sustained for 12 months among those who achieved VS during the first year of follow-up. Unadjusted odds ratios and adjusted odds ratios (aOR) with 95% confidence intervals (CI) are presented for each model. The sex at birth, age, race/ethnicity and site of care type were selected *a priori* and included in all models. In addition to *a priori* variables, predictors were added to the models based on the statistical significance (*P* < 0.05) of the association during bivariate analyses. Final models were chosen based on how the models fit the data using the Hosmer and Lemeshow goodness-of-fit test and assuming the principle of parsimony. All analyses were performed using SAS software, version 9.4 (copyright SAS Institute, Inc., Cary, NC, USA).

## Results

Newly diagnosed participants comprised 5.5% (455/8302) of DC Cohort enrolees. As shown in Table 1, they were mostly Black (69%), male (79%), men who have sex with men (60%), and were a median age of 33 years (IQR 25, 45). Most resided in DC (69%), were on public insurance (53%), and had stable housing (78%), while a lower proportion was employed (35%). Less than half had ever smoked (40%); about a quarter had ever used illicit substances (24%) or ever abused alcohol (24%). At enrolment, the median duration of HIV diagnosis was 4.9 months (IQR 2.3, 7.7), the median nadir CD4 count was 346 cells/μL (IQR 224, 494), median log HIV RNA was 4.08 copies/mL (IQR 2.04, 4.76), and 38% had an AIDS diagnosis.

**Table 1.** Demographic and clinical characteristics^a^, by retention in care (RIC)^b^ status, among individuals newly diagnosed with HIV, DC Cohort, 2011–2016

	Total	Retained in care	Not retained in care
	*N* = 455	*n* = 419	*n* = 36
Characteristic^a^	*N* (%)	*N* (%)	*N* (%)
Age, median (IQR)	33.0 (25.0, 45.0)	34.0 (25.0, 45.0)	29.0 (24.5, 43.5)
Sex at Birth			
Male	358 (78.68)	328 (78.28)	30 (83.33)
Female	97 (21.32)	91 (21.72)	6 (16.67)
Current sex			
Male	352 (77.36)	322 (76.85)	30 (83.33)
Female	95 (20.88)	89 (21.24)	6 (16.67)
Transgender: male-to-female	6 (1.32)	6 (1.43)	0 (0.00)
Transgender: female-to-male	2 (0.44)	2 (0.48)	0 (0.00)
Race/ethnicity			
NH Black	313 (68.79)	288 (68.74)	25 (69.44)
NH White	77 (16.92)	67 (15.99)	10 (27.78)
Hispanic	47 (10.33)	46 (10.98)	1 (2.78)
Other/Unknown	18 (3.96)	18 (4.30)	0 (0.00)
State of residence			
District of Columbia	313 (68.79)	285 (68.05)	28 (77.78)
Maryland	105 (23.08)	100 (23.87)	5 (13.89)
Virginia	33 (7.25)	30 (7.16)	3 (7.25)
Other	4 (0.88)	4 (0.95)	0 (0.00)
Transmission risk			
MSM	273 (60.00)	248 (59.19)	25 (69.44)
IDU	12 (2.64)	11 (2.63)	1 (2.78)
Heterosexual	141 (30.99)	132 (31.50)	9 (25.00)
Perinatal	14 (3.08)	14 (3.34)	0 (0.00)
Unknown	15 (3.30)	14 (3.34)	1 (2.78)
Insurance status (baseline)			
Public	241 (52.97)	224 (53.46)	17 (47.22)
Private	178 (39.12)	163 (38.9)	15 (41.67)
Other/Unknown	36 (7.91)	32 (7.64)	4 (0.88)
Housing status			
Permanent/Stable	355 (78.02)	332 (79.24)	23 (63.89)
Homeless/Temporary	47 (10.33)	40 (9.55)	7 (19.44)
Other/Unknown	53 (11.65)	47 (11.22)	6 (16.67)
Employment status			
Employed	160 (35.16)	147 (35.08)	13 (36.11)
Unemployed	101 (22.20)	90 (21.48)	11 (30.56)
Other/Unknown	194 (43.64)	182 (43.44)	12 (33.33)
Current or past smoking (Yes)	183 (40.22)	167 (39.86)	16 (44.44)
Current or past substance use(Yes)	109 (23.96)	100 (23.87)	9 (25.00)
Current or past alcohol abuse (Yes)	108 (23.74)	99 (23.63)	9 (25.00)
Median HIV duration in months (IQR)	4.92 (2.26, 7.67)	4.92 (2.26, 7.67)	6.07 (2.18, 8.51)
AIDS diagnosis^c^	172 (37.80)	136 (32.46)	5 (13.89)
Nadir CD4 at enrolment (median, IQR)	346 (224, 494)	344 (220, 488)	450 (262, 623)
log HIV RNA at enrolment (median, IQR)	4.08 (2.04,4.76)	4.09 (2.11,4.77)	3.86 (U,4.73)
Site type			
CBO	208 (45.71)	192 (45.82)	16 (44.44)
Hospital	247 (54.29)	227 (54.18)	20 (55.56)
Calendar year of diagnosis			
2010	50 (10.99)	44 (10.50)	6 (16.67)
2011	105 (23.08)	96 (22.91)	9 (25.00)
2012	89 (19.56)	79 (18.85)	10 (27.78)
2013	75 (16.48)	69 (16.57)	6 (16.67)
2014	63 (13.85)	62 (14.80)	1 (2.78)
2015	69 (15.16)	65 (15.51)	4 (11.11)
2016	4 (0.88)	4 (0.95)	0 (0.00)

NH, non-Hispanic; IDU, male or female injection drug user; IQR, interquartile range; MSM, men who have sex with men. Other race groups include those of multiple race groups and unknown.

a Characteristics measured at the time of enrolment.

b Retention in care is defined as having at least two HIV care visits at least 90 days apart in the 12 months post-enrolment.

c Opportunistic infections and CD4 counts <200 cells/μl or CD4% <14.

**Fig. 1.**
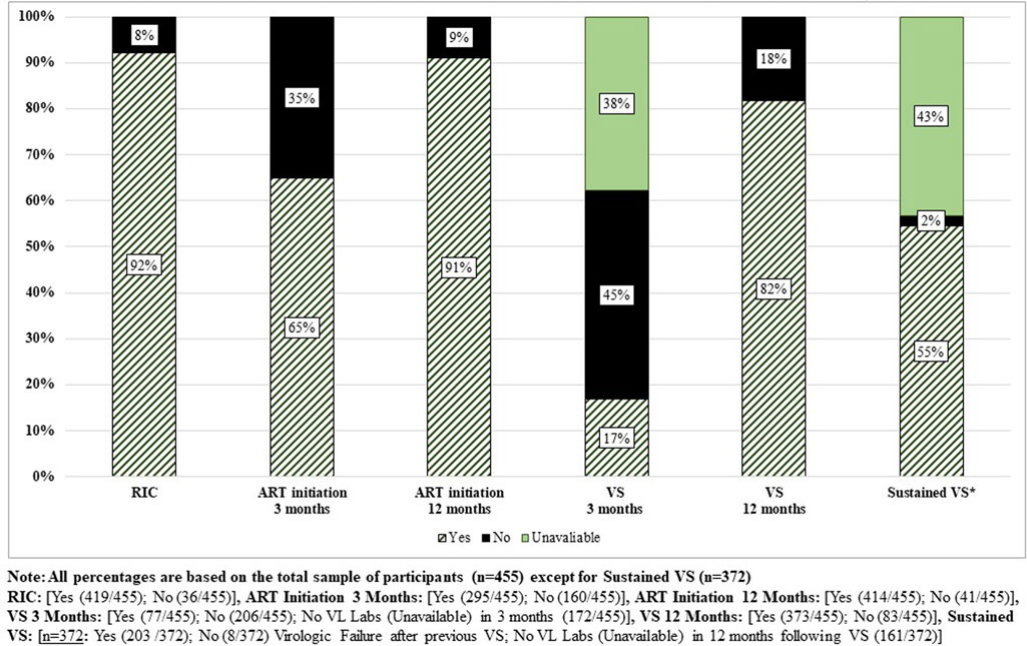
Proportion of newly diagnosed PWH achieving HIV care continuum outcomes, DC Cohort, 2011–2016, *n* = 455.

At 12 months after enrolment, 92% of newly diagnosed PWH were RIC. By 3 months post-HIV diagnosis, 65% had initiated ART; by 12 months post-diagnosis, this proportion increased to 91%. The proportion of participants with VS was 17% and 82% by 3- and 12- months post HIV diagnosis, respectively, and of those with VSV at 12 months, 55% sustained VS for an additional 12 months (Fig. 1).

As shown in Tables 2 and 3, multivariable LR analysis revealed no differences by sex, age or race for RIC, ART initiation, VS and sustained VS. Compared to those without a history of AIDS-defining diagnoses, those with an AIDS diagnosis were three times more likely to be RIC (aOR 2.99; 95% CI 1.13–7.88) and more than two and half times more likely to start ART by 3 months (aOR 2.58; 95% CI 1.61–4.12); they were also almost five times more likely to attain VS by 12 months (aOR 4.87; 95% CI 1.69–14.03).

**Table 2.** Summary of logistic regression Analysis for factors associated with retention in care and ART Initiation

Characteristic^a^	RIC^b^		ART initiation by 3 months		ART initiation by 12 months	
	*N* = 455		*N* = 455		*N* = 455	
	aOR (95% CI)	*P*-value	aOR (95% CI)	*P*-value	aOR (95% CI)	*P*-value
Age	1.07 (0.83–1.37)	0.6049	0.95 (0.82–1.10)	0.4854	0.83 (0.66–1.03)	0.0920
Sex at birth						
Male	Ref		Ref		Ref	
Female	1.56 (0.61–3.98)	0.3507	0.73 (0.45–1.20)	0.2182	0.83 (0.38–1.84)	0.6522
Race/ethnicity						
Other	Ref		Ref		Ref	
NH Black	0.93 (0.43–2.00)	0.8601	0.75 (0.48–1.18)	0.2110	0.72 (0.33–1.56)	0.4044
Substance use: ever *vs*. never	–		0.56 (0.35–0.89)	0.0133	–	
Alcohol abuse: ever *vs*. never	–		–		0.71 (0.33–1.51)	0.3689
HIV duration in months	0.95 (0.85–1.06)	0.3499	–		–	
AIDS diagnosis^c^ (Yes)	2.99 (1.13–7.88)	0.0268	2.58 (1.61–4.12)	<0.0001	4.87 (1.69–14.03)	.0034
Site Type: CBO *vs*. Hospital	1.13 (0.56–2.28)	0.7328	0.77 (0.52–1.16)	0.2136	1.55 (0.77–3.14)	0.2223

aOR, adjusted odds ratio; CI, confidence interval.

a Characteristics measured at the time of enrolment.

b Retention in care is defined as having at least two HIV care visits at least 90 days apart in the 12 months post-enrolment; ART initiation was defined as having been prescribed any antiretroviral medication was assessed at 3- and 12-months following HIV diagnosis date.

c Opportunistic infections and CD4 counts <200 cells/μl^3^ or CD4% <14.

**Table 3.** Summary of logistic regression analysis for factors associated with VS at 3 months, VS at 12 months and sustained viral suppression

Characteristic^a^	Viral suppression at 3 months^b^^,^^c^		Viral suppression at 12 months^b^^,^^c^		Sustained viral suppression^b^^,^^d^	
	*N* = 283		*N* = 455		*N* = 347	
	aOR (95% CI)	*P*-value	aOR (95% CI)	*P*-value	aOR (95% CI)	*P*-value
Age	1.03 (0.85–1.25)	0.7663	1.00 (0.84–1.19)	0.9808	1.14 (0.89–1.46)	0.3009
Sex at birth						
Male	Ref		Ref		Ref	
Female	1.14 (0.58–2.25)	0.6949	0.92 (0.51–1.64)	0.7761	0.91 (0.42–1.99)	0.8211
Race/Ethnicity						
NH Black	1.09 (0.61–1.96)	0.7627	0.66 (0.37–1.16)	0.1493	0.55 (0.25–1.22)	0.1436
Other	Ref		Ref		Ref	
AIDS diagnosis^e^ (Yes *vs*. No)	1.10 (0.63–1.92)	0.7319	2.59 (1.40–4.80)	0.0024	0.79 (0.40–1.57)	0.5053
Site Type: CBO *vs*. Hospital	0.61 (0.36–1.06)	0.0782	1.25 (0.76–2.04)	0.3787	0.70 (0.36–1.35)	0.2865

aOR, adjusted odds ratio; CI, confidence interval.

a Characteristics measured at the time of enrolment.

b VS is defined as having at least one HIV RNA laboratory results <200 by 3 and 12 months after diagnosis; sustained VS is defined as no unsuppressed HIV RNA measure during one year of follow-up after achieving VS 12 months post-diagnosis .

c The number of participants in each model varies according to the HIV RNA laboratory results available at different times. At 3 months post-diagnosis, 283 participants had HIV RNA laboratory results; at 12 months all participants (*N* = 455) had HIV RNA results; 347 participants had HIV RNA results available to calculate sustained viral suppression after 12 months of diagnosis.

d Viral suppression sustained for 12 months among those who achieved VS during the first year of follow-up.

e Opportunistic infections and CD4 counts <200 cells/μl or CD4% <14.

## Discussion

DC Cohort participants recently diagnosed with HIV met the 2020 WHO care continuum outcome indicator goals of 90% on ART and 90% virally suppressed [3] by 12 months after their HIV diagnosis. More than 90% of newly diagnosed PWH were retained in care 12 months after DC Cohort enrolment, and at least two-thirds of DC Cohort participants recently diagnosed with HIV had sustained VS. These findings demonstrate that although most PWH were retained in care, not all individuals were prescribed ART soon after diagnosis, and it took several months for most individuals to start treatment. While we did not have data on the reasons for delayed time to ART in our analysis, some possible explanations include delay in securing insurance coverage, needing time to process an HIV diagnosis, concerns about treatment side effects [20], and provider hesitancy to prescribe due to concerns about adherence [21]. Our data include periods when practices for prescribing ART regardless of CD4 count, had not yet been widely accepted.

Over a third of newly-diagnosed DC Cohort participants from 2011–2016 did not have an HIV RNA viral load result within 3 months of diagnosis, and among the 283 participants that did, only 28% were virally suppressed. This improved to 82% at 12 months. Data from 2011–2015 reported by the DC Health in the 2017 surveillance report [22] revealed that 81.4% of patients were linked to care within 3 months, and 59.7% were suppressed at 12 months, comparable to our 12-month results. *VS* status at three months from the 2011–2015 surveillance data was not reported.

An analysis comparing demographic and clinical characteristics of DC Cohort participants, compared with Washington DC residents with HIV identified with surveillance data, was previously undertaken [23]. The DC Cohort includes individuals receiving HIV care in Washington, DC, who reside in DC and the surrounding metropolitan areas, which include parts of Maryland and Virginia. Approximately 71% of DC Cohort participants reside in DC. The Cohort participants represent a population that is retained in care, and there are some demographic and clinical differences from the general population. In the previous evaluation of surveillance data, there were 12 964 known people living with HIV in DC at the end of 2016. Of those individuals, 40.1% were DC Cohort participants. Compared with nonparticipants, participants were less likely to be male (68.0% *vs.* 74.9%, *P* < 0.001) and more likely to be Black (82.3% *vs.* 69.5%, *P* < 0.001) and have a heterosexual contact HIV transmission risk (30.3% *vs.* 25.9%, *P* < 0.001). DC Cohort participants were also more likely to have ever been diagnosed with stage 3 HIV disease (59.6% *vs.* 47.0%, *P* < 0.001), have a CD4 <200 cells/μl in 2017 (6.2% *vs.* 4.6%, *P* < 0.001) and to be virally suppressed in 2017 (61.4% *vs.* 50.5%, *P* < 0.001) [23]. We do not have a recent comparison between recently diagnosed individuals within the DC Cohort *vs.* those living in DC identified through surveillance data. However, we believe the cohort to be representative of individuals receiving care within Washington DC, since we have enrolled a large proportion of individuals receiving care at the largest HIV clinics.

We found that more advanced HIV was associated with improvements in most of the outcomes evaluated. Those with an AIDS diagnosis at enrolment were approximately twice as likely to be retained in care, to start ART by the third month post-diagnosis and have at least a virally suppressed lab by 12 months after diagnosis. These findings are similar to other large cohorts where a large number of newly diagnosed participants presented with low CD4 counts at diagnosis and at the start of ART [24, 25]. Our results suggest an urgency among providers to promptly treat those with more advanced HIV disease, and also may reflect that patients are more likely to seek care and accept ART when they are ill. However, of equal concern is that patients without AIDS-defining criteria were not started as quickly on ART. This could represent a missed opportunity to prevent the development of HIV reservoirs and reduce underlying inflammation—one of the key rationales for starting ART as early as possible and without consideration of baseline CD4 count or HIV RNA level.

Closer monitoring or more rapid treatment initiation may also explain why those with an AIDS diagnosis were more likely to attain VS compared with those patients with less advanced disease at diagnosis. Additionally, providers may be less likely to immediately treat individuals with high CD4 or low HIV RNA [26]. DC Health has scaled up HIV testing efforts to capture undiagnosed PWH; however, providers and public health officials continue to face challenges due to stigma, complacency about HIV, high migration patterns, poor access to health care, poverty and unemployment [2, 27, 28].

## Strengths/limitations

The linkage with the DC Health HIV/AIDS surveillance system is a major strength of this analysis. Merging data from the DC Cohort with those collected by mandatory reporting to the local surveillance system improves completeness and accuracy of HIV and AIDS diagnoses and provides additional laboratory results that have been collected at clinics and hospitals other than the enrolling DC Cohort site [16]. The DC Cohort study has a consent rate of over 80% of those eligible patients approached and enrols patients seeking HIV care in 14 major clinics in the District. Although there are limitations in the representativeness of the DC Cohort population to all people with HIV in Washington, DC [23] it is a data source that is extremely useful for describing HIV outcomes within DC.

Some of the limitations of this study are due to the nature of the DC Cohort study and DC Health surveillance data collection protocols. All patients included in this study were participants in the DC Cohort and already established in care, precluding assessment of linkage to care (an element of the HIV Care Continuum). Although we were able to assess RIC and other outcomes of the care continuum, we could not determine the proportion of newly diagnosed individuals within all of DC that were linked to care, as not all clinics providing HIV care in DC are part of the DC Cohort. Lastly, the most recent linkage with DC Health before this analysis occurred in 2018. This analysis required at least one year of follow-up data; therefore, patients had to be enrolled by 2016. We are working to establish more rapid data linkage protocols to decrease the delay in using linked data.

## Conclusions

Despite the limitations of this study, this analysis is useful to improve our understanding of persons with recently diagnosed HIV infection, implement the process indicators of the HIV care continuum and assess VS as an outcome indicator in this population. This analysis demonstrates the importance of early HIV testing and timely linkage to care. Prompt identification and RIC of individuals with HIV are crucial steps to ensuring better clinical outcomes and preventing new HIV infections. Additionally, same-day ART initiation is becoming increasingly utilised among newly diagnosed PWH regardless of the patient's HIV stage at diagnosis [29].

## Data Availability

De-identified data available upon written request.

## Code availability (software application or custom code)

Analytic code available upon written request.
